# Effect of SiO_2_ Nanoparticles on PNVCL Polymerization and Molecular Weight Control

**DOI:** 10.1021/acsomega.5c06375

**Published:** 2025-09-12

**Authors:** Arthur M. Gabriel, Maria L. L. Sierra e Silva, Emerson R. de Camargo

**Affiliations:** † Interdisciplinary Laboratory of Electrochemistry and Ceramics (LIEC), Department of Chemistry, 67828 Federal University of São Carlos (UFSCar), São Carlos 13565-905, Brazil; ‡ Nanotechnology National Laboratory for Agriculture, Embrapa Instrumentation, São Carlos 13560-970, Brazil

## Abstract

This study investigates the use of SiO_2_ nanoparticles as functional modifiers in the polymerization of poly­(*N*-vinylcaprolactam) (PNVCL), aiming to tailor the polymer’s molecular weight and physicochemical and rheological behaviors. The incorporation of nanoparticles into polymerization systems has emerged as a promising strategy to modulate the reaction kinetics and polymer chain growth. In this work, SiO_2_ nanoparticles (∼18 nm) were synthesized via the Stober method, incorporated into the radical polymerization reaction of PNVCL at a concentration ranging from 0.5 to 3.0%, and recovered post synthesis. Gel permeation chromatography results revealed that increasing the nanoparticle concentration led to a notable rise in the weight-average molecular weight from 33 to 70 kDa. Rheological analysis revealed that the materials exhibited a predominantly viscous behavior (*G*″ > *G*′) both below and above the lower critical solution temperature, suggesting incomplete gelation transitions. These findings demonstrate that SiO_2_ nanoparticles can act as polymerization modulators, enabling control over molecular weight distribution and viscoelastic properties, with relevance for thermoresponsive systems for drug delivery and related biomedical technologies.

## Graphical abstract

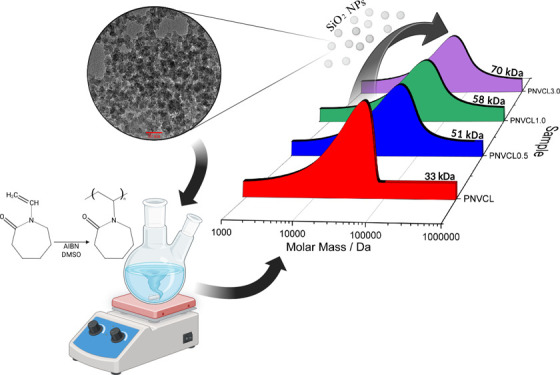

## 1 Introduction

Hydrogels are cross-linked three-dimensional polymeric networks that have the ability to absorb significant amounts of water, swell, and retain moisture, while the structure is maintained. A smart class of these hydrogels are called responsive hydrogels due to their ability to alter their physical and chemical properties in response to environmental stimuli, including temperature, pH, ionic strength, light sources, and even magnetic fields.^1,2^ This sensitivity stems from the intrinsic polymer chemistry and the functional groups interacting with the surrounding environment. An example is the pH-responsive hydrogels that contain ionizable functional groups, like carboxyl or amine groups, and are able to swell or contract depending on the pH of the environment, which changes the degree of ionization and the electrostatic interactions within the polymer network.^3^

A particular class of responsive hydrogels, thermoresponsive hydrogels, have gained specific interest for their ability to respond to temperature changes, making them highly applicable in biomedicine, particularly for controlled drug-release systems. These hydrogels are typically synthesized using polymers containing lower critical solution temperature (LCST) behavior, where the polymer network remains hydrated and swollen below the LCST but deswells and expels water above this temperature.^4,5^ For example, poly­(*N*-vinylcaprolactam) (PNVCL) is a commonly used thermoresponsive polymer due to its biocompatibility and LCST close to the physiological temperature. PNVCL thermoresponsiveness mechanism involves the balance of hydrophilic and hydrophobic interactions: at lower temperatures, hydrogen bonding between the polymer and water molecules predominates, maintaining the hydrogel swollen. However, above the LCST, hydrophobic interactions among polymer chains become dominant, causing the network to collapse and expel water. This transition allows for tailored applications in drug delivery, where the release of therapeutic agents can be controlled by modulating the ambient temperature, providing a highly efficient method for targeted and localized treatment.^6,7^

Research on responsive polymers, including PNVCL, has led to notable advances in materials science, particularly in designing polymers with tunable properties for targeted biomedical uses.^8–10^ For example, Ribeiro et al. developed injectable PNVCL nanocomposites for localized delivery of hydrophobic (naringin) and hydrophilic (doxorubicin) drugs. The developed system was capable of releasing the drugs for 7 days under physiological conditions and demonstrated selective cytotoxicity toward bladder carcinoma cells while preserving the viability of healthy fibroblasts (L929).^11^ Indulekha et al. developed thermoresponsive chitosan-*g*-PNVCL polymer gels for on-demand transdermal delivery of 4-acetamidophenol and etoricoxib. The material demonstrated transdermal release capacity and biocompatibility in in vivo assays.^12^ These results demonstrate the potential of thermoresponsive systems, as made by PNVCL, in biological applications due to their injectability, responsiveness, and ability to modulate release profiles and hydrophilic and hydrophobic molecular loadings.

An important feature for the application of PNVCL-based systems is the tunability of their molecular properties, particularly molecular weight and viscoelastic profile, which directly affect their sol–gel transition, swelling kinetics, and drug-delivery capabilities.^13–15^

Controlling the polymerization kinetics and molecular architecture of PNVCL is essential for tailoring its properties for specific applications. In this context, nanomaterials emerge as promising additives and polymerization modifiers.^16,17^ Among them, SiO_2_ nanoparticles are particularly attractive due to their high surface area, colloidal stability, and ease of surface functionalization.^18^ Functionalized silica nanoparticles can influence chain propagation and termination events through surface-mediated interactions with monomers and initiators.^19–21^ Depending on the surface chemistry and concentration, nanoparticles can slow or accelerate polymerization reactions and consequently modulate the molecular weight and polymer architecture.^18,22–24^

Studies report that the use of silica nanoparticles, especially those measuring 10–50 nm, can induce confinement effects, alter radical diffusion rates, or participate in surface-initiated polymerization, altering chain growth kinetics.^25,26^ These effects have been exploited to obtain polymers with tailored chain lengths, reduced gelation times, or enhanced mechanical properties.^27^ Furthermore, the inorganic and stable nature of silica presents great potential for recovery and reuse, which also contribute to the sustainability of such modified polymerization systems.^9,26^ Although the role of nanoparticles as catalysts and in the formation of nanocomposites is well established, their influence on the polymerization kinetics of thermoresponsive polymers such as PNVCL remains unexplored. Understanding how nanoparticle interactions and concentration affect molecular weight development in PNVCL systems could enable the rational design of responsive materials with precise structural control.

The influence of molecular mass on the polymer structure and properties is a critical factor in determining drug loading and delivery capacities, especially when considering its application in controlled release systems. High-molecular-weight polymers often form more extensive and entangled networks due to increased chain entanglements and intermolecular interactions, leading to denser and more stable hydrogel structures.^28^ These hydrogels typically exhibit enhanced mechanical strength, swelling capacity, and sustained drug-release profiles compared with lower-molecular-weight counterparts. Additionally, higher-molecular-weight polymers offer greater drug-loading capacity as they can accommodate a larger number of drug molecules within their matrix, thereby improving drug-delivery efficiency and therapeutic outcomes.^29,30^ Studies have shown that variations in the molecular mass significantly impact the cross-linking density, mesh size, and swelling behavior of hydrogels, consequently affecting their drug loading and delivery capacities.^31^ Thus, understanding the relationship between molecular mass and polymer properties is essential for optimizing hydrogel formulations for effective applications, such as drug-delivery systems.^32–35^

The aim of this study was to provide a novel approach by utilizing SiO_2_ nanoparticles to modulate PNVCL molecular weight during polymerization, a strategy that, to our knowledge, has not been previously reported. This work also brings novelty by the combination of multiple analytical techniques to semiquantitatively compare the determining trends in molecular weights, bringing insights into the role of nanomaterials in polymeric synthesis.

## 2 Experimental Section

### 2.1 Silica Nanoparticle Synthesis

The spherical silica nanoparticles were synthesized following a previous methodology described by our group based on the Stöber method.^36^ Initially, 0.02 mol of tetraethyl orthosilicate (TEOS) (Sigma-Aldrich 98%) was added to 1.71 mol of anhydrous ethanol (Synth 98.9%); 0.03 mol of ammonium hydroxide (NH_4_OH) (Synth 27%) was added to the solution and the system were kept under constant magnetic stirring (800 rpm) for 24 h at room temperature. Later, the nanoparticles were washed by four consecutive steps of centrifugation at 9000 rpm for 10 min using anhydrous ethanol and dried at 60 °C for 12h.

### 2.2 PNVCL Synthesis

The polymer was synthesized by a radical polymerization procedure described by our group.^10^ For this, 0.0538 mol of the *N*-vinylcaprolactam (NVCL) monomer (Sigma-Aldrich 98%) was dissolved in 0.38 mol of anhydrous dimethyl sulfoxide (DMSO) (Synth 99.9%) and heated to 70 °C into a jacketed reactor under a nitrogen atmosphere for 15 min. Later, 1.02 mmol of the initiator 2,2-azobisisobutironitrile (Dupont) was dissolved in 0.16 mol of DMSO and added dropwise in the monomer solution trough an addition funnel. The initiator was previously purified by recrystallization in methanol. The reaction system was kept under magnetic stirring for 4 h. The obtained polymer was precipitated using ultrapure water and purified through 4 days of aqueous dialysis using a membrane tube with a *M*_w_ cutoff of 3500 Da. Then, it was filtered using a sintered glass filter to remove the catalyst and dried in an oven at 60 °C for 24 h. The nanoparticles were used as the polymerization catalyst, and they were added at 0.5, 1.0, and 3.0% (relative to the monomer’s mass) to the reaction medium prior to the initiator’s addition. To determine the kinetic parameters of polymerization, aliquots were taken during synthesis at predetermined times. The synthesis of each sample was performed in triplicate.

### 2.3 Characterizations

Structural characterization of the nanoparticles and the polymers was conducted by Fourier transform infrared spectroscopy using attenuated total reflectance (FTIR-ATR) on a Bruker Vertex 70 spectrometer, with the spectra recorded between 400 and 4000 cm^–1^. FTIR-ATR was also used to acquire the spectra of the aliquots taken during polymerization. Ten aliquots were taken at predetermined times during the reaction and transferred to ice-cooled tubes containing a polymerization inhibitor (hydroquinone) to quench the reaction. The monomer conversion was determined by monitoring the decrease in the absorbance of the vinyl C=C stretching band at 1659 cm^–^^1^, which is characteristic of the NVCL monomer. The intensity of this band was normalized using an internal reference peak (C–N at 1429 cm^–^^1^), and the conversion was calculated based on the area ratio between these peaks, providing a semiquantitative assessment of polymerization progress over time.

The nanoparticle morphology and size were visualized by transmission electron microscopy (TEM) on a JEOL JEM-3010 microscope operating at 300 kV equipped with a GATAN Multi-Scan CCD camera, using secondary electrons to form the images. The samples were prepared by dispersing the nanoparticles in deionized water (1 mg/mL), and the suspension was sonicated followed by drop-casting it onto a copper grid and allowed to dry at room temperature.

Dynamic light scattering (DLS) was used to visualize the polymers’ LCST. The measurements were taken on a Malvern Zetasizer Nano-ZS Zen 3600 at an angle of 173° and a wavelength of 633 nm. Polymer solutions were prepared (10 mg/mL) in deionized water (pH = 7) and stored at 4 °C for 24 h to ensure complete solubilization prior to measurement. The measurements were carried out at room temperature and also varied between 25.0 and 38 °C, with each measurement being carried out in triplicate and left in equilibrium for 2 min at each point. The LCST was attributed to the temperature at which a dramatic change in the hydrodynamic diameter was observed.

DLS was also used as a semiquantitative method to estimate molecular masses. Measurements were conducted in triplicate at 25 °C. The hydrodynamic diameter (*D*_h_) was determined from the intensity-weighted size distribution between 5 and 30 nm, and the molar mass was estimated by the correlation between *D*_h_ and the radius of gyration (*R*_g_) under the assumption of a random coil conformation in a good solvent. The radius of gyration was approximated as *R*_g_ = *D*_h_ × 0.75. Subsequently, the molecular mass was calculated using the empirical relationship $R_{g}=2.94\times 10^{-2}{M_{w}}^{0.54}$ (1) as reported for flexible polymers in dilute solutions.^37,38^ This indirect method does not provide absolute molecular weight values and is sensitive to assumptions such as chain conformation, solvation state, and absence of aggregation. Nevertheless, it offers a useful comparative perspective on relative molecular mass differences across samples.

The glass transition temperature (*T*_g_) for the polymers was determined by differential scanning calorimetry using a Netzsch DSC 404C calorimeter between 25 and 240 °C and an aluminum crucible at a constant heating/cooling rate of 10 °C/min with a nitrogen flux of 0.50 cm^3^/min.

Rheological analysis was performed to evaluate the viscoelastic properties of the polymers at 25 and 37 °C. Solutions with a concentration of 20% were characterized on an Anton-Paar MCR 302, equipped with a parallel geometry plate with a 25 mm diameter and a gap of 0.5 mm. The region of linear viscoelasticity was determined by varying the deformation from 0.001 to 100% at a frequency of 1 Hz, and a time sweep analysis was performed using a strain of 1.0% and a frequency of 1 Hz during a 10 min interval.

The changes in viscosity with temperature of the polymers were analyzed using an AMVn automated microscope (Anton-Paar). Polymeric dispersions with a concentration of 10 mg/mL in distilled water, heated from 32.0 to 38.0 °C, were analyzed, and the viscosities were measured every 0.5 °C at angles 70° and −70°. The viscosimetric molar masses were also determined using solutions of each sample, where the reduced viscosity was calculated by the Huggins equation, and the molar mass was calculated by the Mark–Houkink–Sakurada equation.

The molar mass was evaluated using size exclusion chromatography (SEC). Analysis of 200 μL of the polymer solutions (0.05 g/mL) was performed using tetrahydrofuran (THF) as an eluent at 50 °C at a flow rate of 1 mL/min on a Viscotek HT-GPC (Malvern) instrument with three H-806 M columns (mixed) and a refractive index detector. Calibration was done using narrowly distributed polystyrene standards (500–2,500,000 g/mol).

## 3 Results and Discussion

The nanoparticles synthesized by the Stöber based method were used as molar mass modulator in PNVCL polymerization.^36^ Figure 1 shows the particle’s morphology, and an average size of 18.2 ± 2.5 nm was determined. The size distribution graph is shown in Figure S1. The particles were characterized chemically by FTIR spectroscopy, as shown in Figure S2. The spectra show a peak at 787 cm^–1^ corresponding to the symmetrical stretching vibration of Si–O. The peak at 964 cm^–1^ is attributed to the Si–OH stretching, and the one at 1063 cm^–1^ is attributed to the asymmetric Si–O–Si stretching.^36,39^ The way it was synthesized, without a functionalization step, guarantees that it does not covalently bind to the PNVCL chains during polymerization but rather acts as a modifying agent in the reaction.

**Figure 1:**
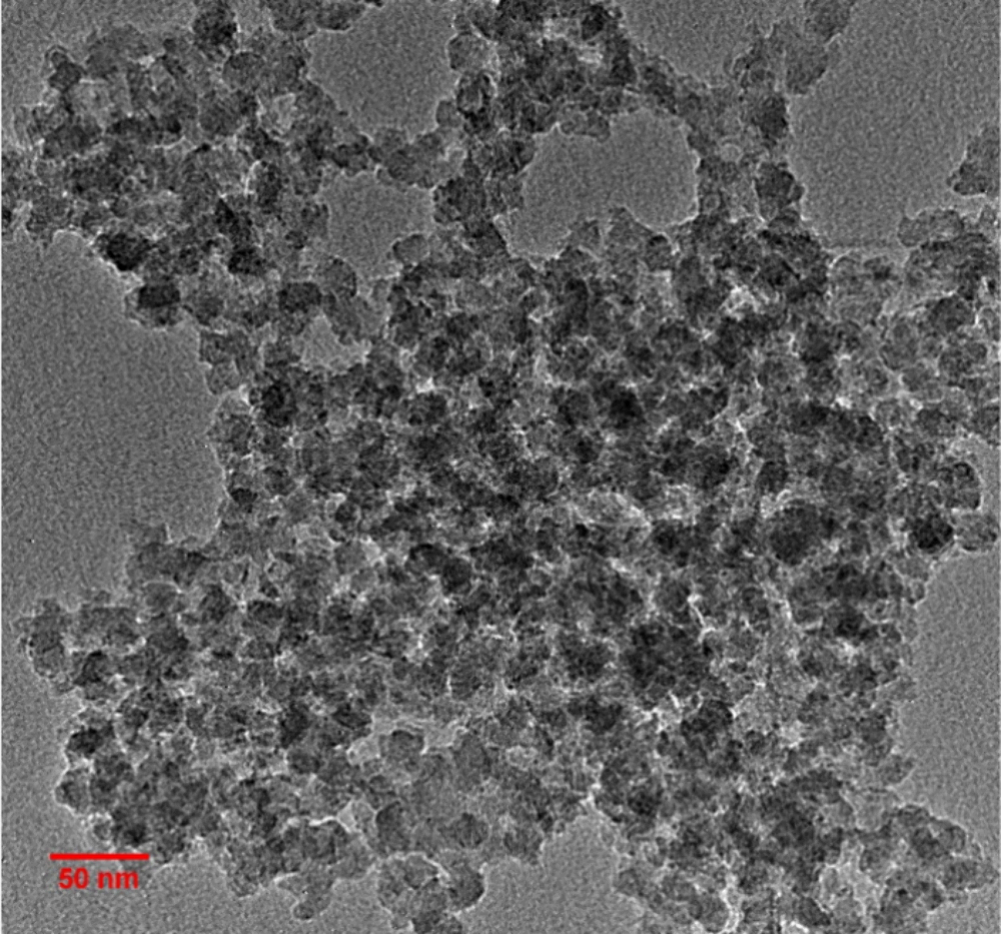
TEM image of SiO_2_ nanoparticles.

The *N*-vinylcaprolactam polymerization follows a free-radical chain propagation mechanism initiated by a thermal radical initiator. Figure S3a depicts the overall reaction. The initiation step (Figure S3b) starts with the decomposition of the initiation, generating free radicals that react with the monomer and form active radical species. The propagation (Figure S3c) occurs as the formed radicals continuously add to additional monomer units, leading to chain growth and extending the polymeric chain. Termination occurs by recombination (Figure S3d), when two growing radicals combine, or by disproportionation (Figure S3e), when a radical is deactivated by a transfer reaction. The balance between these steps determines the final molecular weight and distribution.

The polymers were synthesized by solution polymerization with the addition of none, 0.5, 1.0, and 3.0% of the silica nanoparticles. Figure S4 shows the FTIR spectra revealing the main bands of the polymer, namely, stretching of the C=O and C–N bonds at 1633 and 1429 cm^–1^, out-of-plane vibrations of the CH_2_ group at 1310–1190 cm^–1^, symmetric and asymmetric stretches of the CH_2_ group at 2896 and 2969 cm^–1^, respectively, and the absence of the 1659 cm^–1^ band for the C=C bond. Comparing these spectra, it is seen that PNVCL was formed as a polymer in all reactions. Furthermore, the absence of characteristic silica bands indicates that they were not bound to the polymer chain and were effectively removed from the reaction product. Also, the characteristic spectrum shows that there was no formation of any other product, polymer degradation, or ring opening.

The thermodynamic and kinetic parameters of the polymerization were also defined. The standard synthesis was conducted at different temperatures (60 and 80 °C) to obtain reaction rates and calculate the activation energy. Figure S5 shows the reaction progress under different temperature conditions. It is noted that the reaction occurs more rapidly at higher temperatures, necessitating less time to achieve a higher conversion rate. Figure S6 shows the dependence of the polymerization rate on temperature (Arrhenius Plot). The linear dependence of the speed with 1/*T* (*R* ^2^ = 0.98) is shown. The *E*_a_ of the reaction was calculated, and a value of 126.5 kJ was obtained, a value slightly above the typical *E*_a_ values for polymerizations initiated by thermal decomposition. By possessing a higher viscosity than most solvents, DMSO can contribute to the formation of the complex between the monomer and the initiator, in a way that increases the activation energy, as explained by the cage effect theory.^40–42^

In the context of modified polymerizations, the influence of nanoparticles on the monomer conversion degree, the reaction rate, and the final product molecular weight was evaluated. Figure 2 shows the variation in the conversion rates of each sample as a function of the polymerization reaction time. FTIR-ATR was chosen in this work due to its accessibility, simplicity, and ability to provide monitoring of functional group consumption during the reaction.

**Figure 2:**
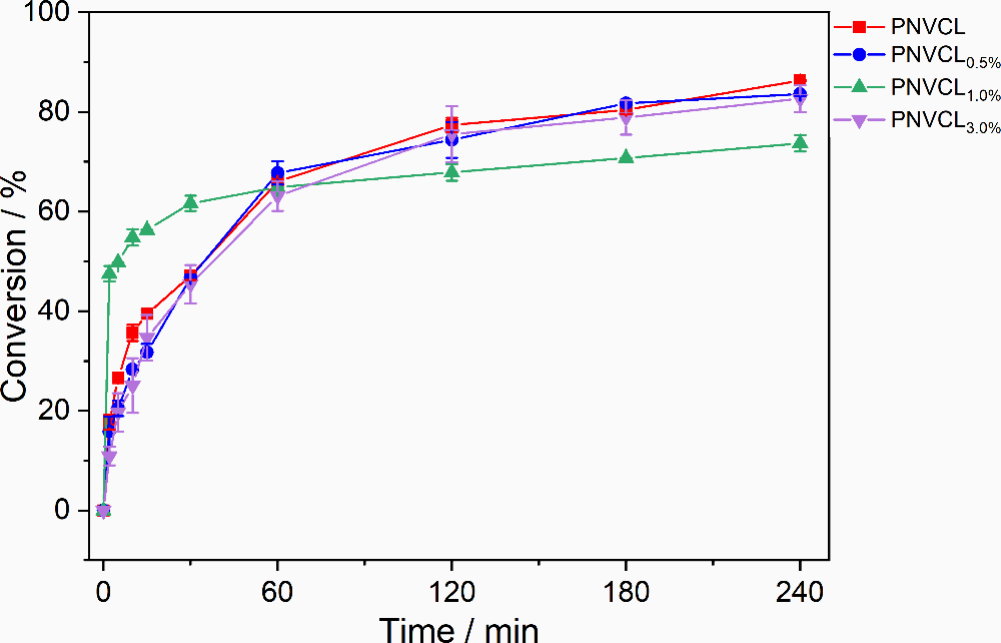
Conversion variation with time of the different polymerization reactions of PNVCL using 0, 0.5, 1.0, and 3.0% silica nanoparticles, 70 °C, 24 h.

A proportional trend in initial reaction rates was observed on increasing the nanoparticle concentration, but a decrease occurred when 3% concentration was used. It is believed that at this concentration, the nanoparticles agglomerated in the reaction medium, reducing their interaction capacity. Zeta potential measurements of nanoparticle suspensions at the same concentrations used in polymerization suggest a decrease in colloidal stability at higher concentrations. Zeta potential moduli decreased significantly with increasing concentration from −36.3 mV at 0.5% to −10.7 mV at 3.0%. This trend indicates weaker electrostatic repulsion between particles, suggesting an increased tendency for agglomeration at higher concentrations.^43,44^ These agglomerations may reduce the available surface area and active sites for polymerization, thereby contributing to the observed decrease in the polymerization rate at 3.0%. At the end of the reaction, a reversal of this trend was seen in terms of conversion rates. In the same way that 1% > 0% > 0.5% > 3% directly affects the initial speed, it inversely affects the conversion rate and consequently the molecular weights. Table 1 shows the initial speed for the polymerization reactions.

**Table 1:** Polymerization Initial Speed

sample	*R*_p_ (% min^–1^)
PNVCL	9.21
PNVCL_0.5%_	7.91
PNVCL_1.0%_	23.74
PNVCL_3.0%_	5.45

DLS was used to determine the LCST and estimate the molecular mass. The LCST was attributed to the temperature where a change in *D*_h_ was observed, and the average molecular mass was estimated from eq 1. This technique was primarily used not to determine the molecular weight but to illustrate a trend that supports the hypothesis being investigated. LCST results are shown in Figure 3 and compared to the estimated molecular mass in Table 2.

**Figure 3:**
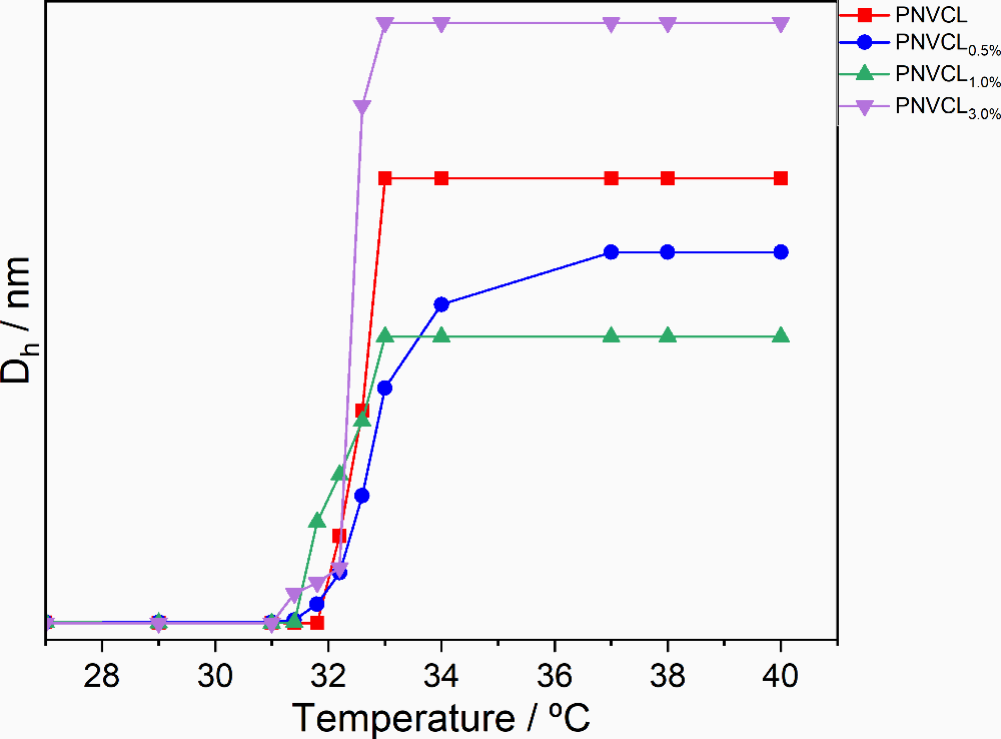
Variation of the hydrodynamic diameter at different temperatures.

**Table 2:** Molecular Weight, LCST, and *T*_g_ of Products

sample	*M*_w_ (kDa)	LCST (°C)	*T*_g_ (°C)
PNVCL	66.0	33.4	183.1
PNVCL_0.5%_	81.5	33	185.8
PNVCL_1.0%_	167.4	32.6	188
PNVCL_3.0%_	195.1	31.8	191.5

These results show a clear trend where the LCST decreases as the molar mass increases, which is also linked to an increase in the amount of silica used. This behavior stems from the complex interactions between hydrophilic and hydrophobic elements within the polymer matrix as the molecular weight changes. Importantly, PNVCL polymers with higher molecular weight exhibit more pronounced hydrophobic segments, which contribute to a reduced LCST.^45^ This phenomenon is influenced by the increased chain length and structural complexity, enhancing the hydrophobic forces within the polymer and leading to phase separation at lower temperatures. These observed correlations align with the literature, further validating the established relationship between the molecular mass, LCST, and polymer behavior.^13,38,46^ Regarding the glass transition temperature, it is also observed that the increase in the molecular mass causes an increase in *T*_g_. DSC curves are shown in Figure S7. This occurs because polymers with higher molecular masses exhibit reduced chain mobility due to entanglement density and increased van der Waals interactions between the longer chains. This restricted molecular motion requires more energy to reach the segmental motion characteristic of *T*_g_, resulting in a high transition temperature.

Viscosimetry was also used to determine the LCST by measuring the dynamic viscosity at different temperatures. Once again, this was used as a tool to illustrate the trends in the properties and to establish the relationship with molecular mass, resulting in the determination of the viscometric molecular weight. Similar to what was observed with DLS, the polymers synthesized with a higher amount of catalyst exhibited a lower LSCT. Figure 4 shows the viscosity changes of the samples at different temperatures.

**Figure 4:**
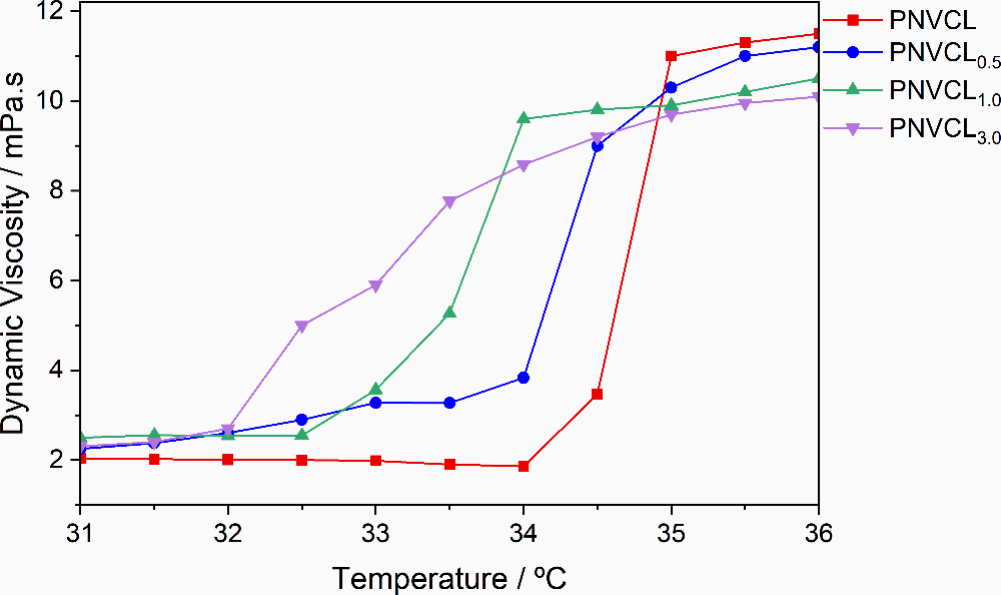
Variation of the dynamic viscosity of the molecules at different temperatures.

The same trend is observed for the viscosimetric molar mass. An increase in mass was observed due to the increase in the amount of nanoparticles used. Table 3 shows this change in the mass of different materials. The possibility to tune the material solution’s viscosity is critical for applications in controlled drug-delivery systems within the human body. The viscosity modulation allows for the optimization of flow properties, ensuring efficient administration and targeted delivery to specific organs and tissues. Manipulation materials with temperature responsive viscosity, especially those that exhibit significant changes near physiological temperatures, can show several advantages. This transition from a low-viscosity state for ease of injection to a high-viscosity state for prolonged retention and adhesion at the site of interest can improve therapeutic efficacy. This behavior is particularly useful in scenarios where localized and sustained drug release is required, such as in wound healing, cancer treatment, or regenerative medicine. Besides that, tailoring the viscosity properties enables better control over drug diffusion rates, enhancing the system’s overall precision and minimizing potential side effects.^47,48^

**Table 3:** Viscosimetric Molar Masses

sample	viscosimetric *M*_w_ (kDa)
PNVCL	67.9
PNVCL_0.5%_	74.9
PNVCL_1.0%_	79.6
PNVCL_3.0%_	88.5

The viscosimetric behavior of the samples is visualized in Figure 5. At 25 °C, where the samples are below the LCST, a direct correlation is observed between viscosity and molecular weight, which can be attributed to the increased hydrodynamic volume and chain entanglement typical of solvated, extended polymer conformations. However, at 37 °C, below the LCST, the samples exhibit a decrease in viscosity with increasing molecular weight. This inverse relationship can be explained by a more effective collapse in higher-molecular-weight polymers, which reduces their effective hydrodynamic contribution and limits interchain entanglement. The predominance of hydrophobic interactions results in chain collapse and a reduced contribution to bulk viscosity. This emphasizes that above the LSCT, systems become less dependent on the chain size and more influenced by globule compactness and aggregation dynamics.

**Figure 5:**
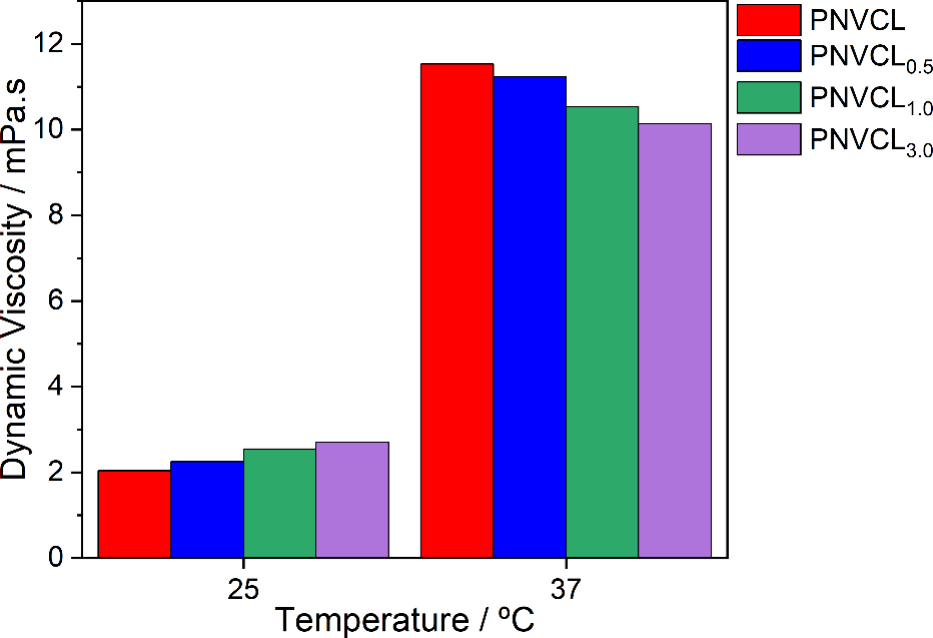
Viscosity of the samples below and above the LCST.

The effect of the molecular weight on the rheological properties was evaluated to visualize the injectability of the material. Thus, storage modulus (*G*′) and loss modulus (*G*″) were analyzed below and above the LCST, as shown in Figure 6. These tests were conducted in the linear viscoelastic region. For hydrogels, the standard rheological behavior is based on the predominance of elastic behavior (*G*′ > *G*″) where the material behaves as a gel or a viscoelastic network below the LCST and begin to exhibit a more fluid or viscous behavior (*G*′ < *G*″) as the temperature increases. However, this was not observed in this study. After heating above the LCST, it was observed that *G*″ remained greater than the storage modulus. This suggests that the material may be in a viscous fluid regime rather than being fully gelled.

**Figure 6:**
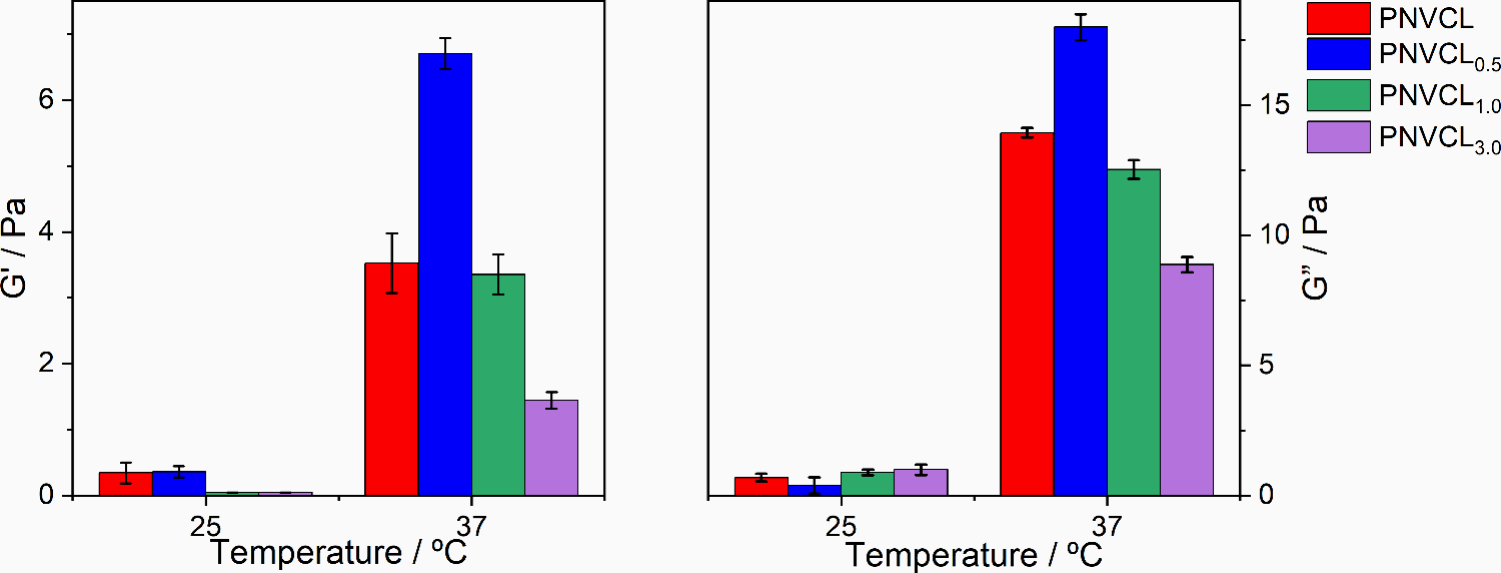
*G*′ and *G*″ of the polymers above and below the LCST.

Although rheological measurements above the LCST did not confirm a complete transition to an elastic state, macroscopic changes in the physical states of the samples were observed. After heating, the solutions formed gel-like, structured phases with reduced fluidity and increased consistency, indicating the occurrence of temperature-triggered molecular rearrangements. This behavior suggests that even in the absence of a network with completely elastic behavior, the system undergoes aggregation and partial association of polymer chains, induced by hydrophobic interactions and chain collapse. Nevertheless, the formation of a more structured phase above the LCST supports the idea of impaired chain interaction, a behavior relevant to biomedical applications that relies on phase transition behavior rather than full gel formation.

Thus, it can be concluded that heating up above the material’s solubility transition is not sufficient to induce a transition to the gelled state. Other authors have also reported that for PNVCL, there is a difference between the solubility transition temperature and the transition to the gelled state, with temperatures above 45 °C being required for the storage modulus to exceed the loss modulus.^35,49,50^

For the material studied, no direct trend was observed between the molecular weights and the storage and loss moduli, suggesting that the relationship between the molecular weight and the rheological properties of the material is neither simple nor linear. However, a relationship between the initial polymerization rate and *G*′ and *G*″ was observed, which can provide valuable insights. This may be attributed to the entanglement of the polymer chains. A higher initial polymerization rate likely leads to more rapid chain formation, resulting in increased entanglement. This entanglement can usually enhance both the elasticity (*G*′) and viscosity (*G*″) of the material as a more entangled network typically leads to higher resistance to flow and increased energy-storage capacity.^51,52^

The molecular weight of the polymers measured by GPC is presented in Table 4, and the curves are shown in Figure 7. The data reveal a trend where the ponderal molecular weight (*M*_w_) and the *z*-average molecular weight (*M*_*z*_) increase with the amount of added silica nanoparticles. This suggests that the presence of the nanomaterial effectively promotes the formation of higher-molecular-weight polymer chains by influencing the kinetics of polymerization. So far, the presented data support the hypothesis that the addition of silica nanoparticles to the polymerization system can effectively modify its properties and that the molecular weight of the synthesized polymers is directly influenced by the amount of nanomaterial added.

**Figure 7:**
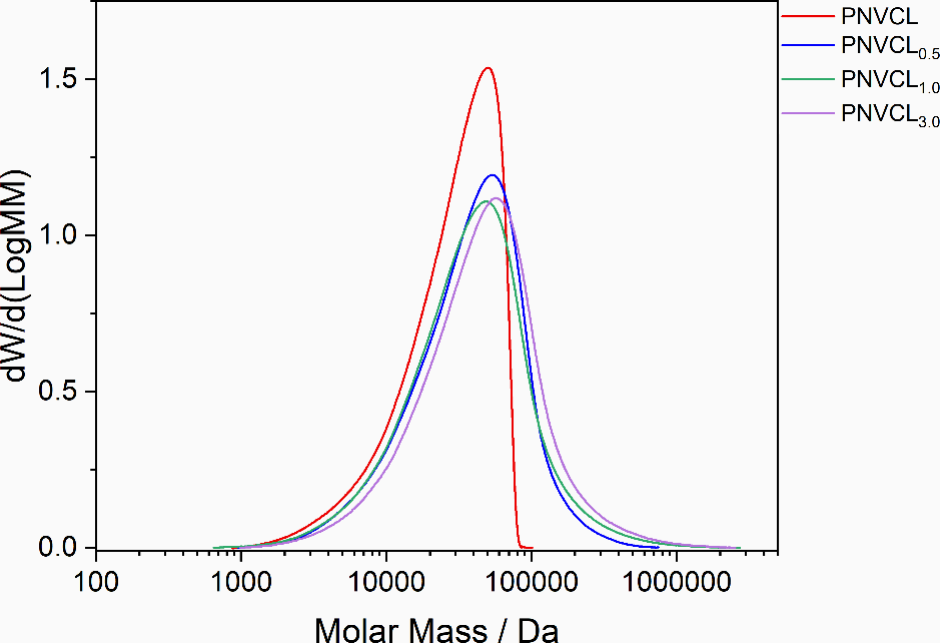
GPC chromatograms of PNVCL samples.

**Table 4:** Molecular Masses Determined by GPC

sample	*M*_n_ (kDa)	*M*_w_ (kDa)	*M*_*z*_ (kDa)	PDI
PNVCL	18.44	33.1	43.6	1.79
PNVCL_0.5%_	23.07	51.3	95.6	2.22
PNVCL_1.0%_	22.16	58.8	182.7	2.65
PNVCL_3.0%_	26.64	70.7	215.7	2.65

The number-average molecular weight (*M*_n_) provides higher emphasis on smaller chains within the distribution. In this study, *M*_n_ values exhibit a nonlinear trend with the increase of nanoparticle concentration. The initial increase from 18.4 kDa (PNVCL) to 23.0 kDa (PNVCL_0.5%_) reveals that the nanomaterial can promote chain growth; however, the small drop to 22.1 kDa (PNVCL_1.0%_) possibly indicates a higher contribution to chain termination. Another linear increase observed to 26.6 kDa (PNVCL_3.0%_) suggests that a higher concentration of nanoparticles turns polymeric chain propagation favorable again. Since this molecular mass is closely related to the average polymer chain length, this variation implies that silica nanoparticles are affecting the balance between initiation, propagation, and termination steps, leading to a more complex molecular weight evaluation. Since *M*_n_ is more sensitive to chain initiation and termination processes, these variations suggest that while the nanoparticles facilitate polymerization, they may also introduce effects such as chain transfer or radical recombination, leading to a complex interplay between chain growth and termination.

The weight-average molecular weight (*M*_w_) gets a higher influence of the larger polymer chains in the distribution. The observed trend shows a continuous increase in the molecular weight with increasing silica concentration from 33.1 kDa (PNVCL) to a final 70.7 kDa (PNVCL_3.0%_). It suggests that there is an effective promotion in the formation of longer polymer chains, likely by stabilizing growing radicals or influencing chain-transfer mechanisms. This increase in M_W_ usually correlates with an enhanced mechanical strength and improved viscoelastic properties in polymer networks. Given that *M*_w_ correlates with solution viscosity, these changes also influence the polymer’s flow properties, as discussed, a key point in applications that require tunable viscosity, like injectable hydrogels. This suggests that the silica addition allows for better mechanical properties tuning, particularly useful for controlled drug-release applications.

The *z*-average molecular weight (*M*_*z*_) is more sensitive to the longest polymer chains in the distribution. The results present a substantial increase in *M*_*z*_ from 43.6 kDa (PNVCL) to 215.7 kDa (PNVCL_3.0%_), which suggests that a fraction of the polymer population consists of longer chains at higher concentrations of nanoparticles. The sharp increase in *M*_*z*_, specially at 1.0 and 3.0% nanoparticle concentrations, indicates that the SiO_2_ nanoparticles facilitate the formation of a set of extremely high-molecular-weight chains, potentially by reducing chain termination rates. Since *M*_*z*_ is directly linked to the polymer’s diffusion properties, the increase suggests that higher nanoparticle concentrations could lead to networks with slower diffusion rates, affecting permeability and release kinetics in applications such as drug delivery.

The results presented highlight the fundamental role of silica nanoparticles in the polymerization process, where different concentrations of this nanomaterial lead to reaction pathways with different kinetic rates. This variation directly influenced the molar mass distribution of the synthesized polymers, demonstrating that the final macromolecular characteristics are highly dependent on the amount of nanomaterial added to the polymerization. The results suggest that the nanoparticles may be accelerating the key stages of the polymerization, initiation, propagation, and termination or playing a stabilizing role in the radical species, strongly modulating the growth and structural properties of the resulting polymer chains. Thus, the potential of this type of reaction in controlling the properties of polymeric materials is verified.

## 4 Conclusions

In this study, we demonstrate that silica nanoparticles can be effectively used as modifier agents in the polymerization reaction of PNVCL, with the advantage of being recoverable at the end of the process. The incorporation of these nanoparticles as a “catalyst” let to substantial differences in the physicochemical properties of the resulting materials. Significant changes in the molecular weight of the polymer were observed, correlating with the amount of SiO_2_ nanoparticles used. Furthermore, the rheological analysis revealed important insights into the material’s behavior, with both storage and loss moduli indicating a pronounced shift in the system’s viscoelastic properties. These findings suggest that SiO_2_ nanoparticles not only impact the polymerization process but also impact the final material’s structure and mechanical characteristics, paving a way for the development of tailored materials with specific rheological properties for various biological applications

## Supplementary Material



## Notes

Size distribution of SiO_2_ nanoparticles; FTIR spectra of SiO_2_ nanoparticles; PNVCL polymerization reaction steps; FTIR spectra of PNVCL products; dependence of reaction conversion on temperature; Arrhenius plot; and DSC curves (PDF)
