# Supplementary material for: Effect of SiO2 Nanoparticles on PNVCL Polymerization and Molecular Weight Control
Source: ACS Omega. 2025 Sep 12;10(37):43141–9. doi: 10.1021/acsomega.5c06375 (PMC12461300; doi:10.1021/acsomega.5c06375)
Supplement: Supplementary file 1 [file ao5c06375_si_001.pdf]

Supporting Information

Effect of SiO_2 Nanoparticles on PNVCL Polymerization and Molecular Weight Control

*Arthur M Gabriel, Maria L L S e Silva, Emerson R de Camargo**

Arthur M Gabriel, Emerson R de Camargo
Interdisciplinary Laboratory of Electrochemistry and Ceramics (LIEC), Department of Chemistry, Federal University of São Carlos (UFSCar), São Carlos, 13565-905 Brazil
*E-mail: camargo@ufscar.br

Maria L L S e Silva
Nanotechnology National Laboratory for Agriculture, Embrapa Instrumentation, São Carlos, 13560-970, Brazil

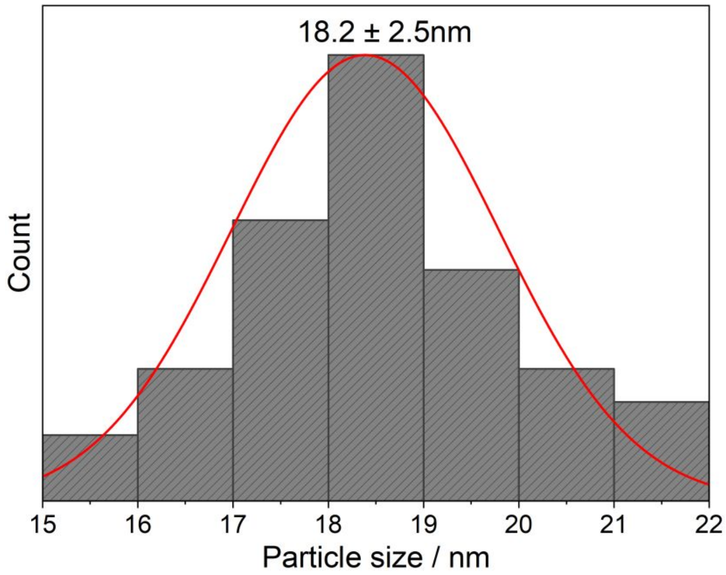

Figure S1. *Size distribution of SiO_2 nanoparticles.*

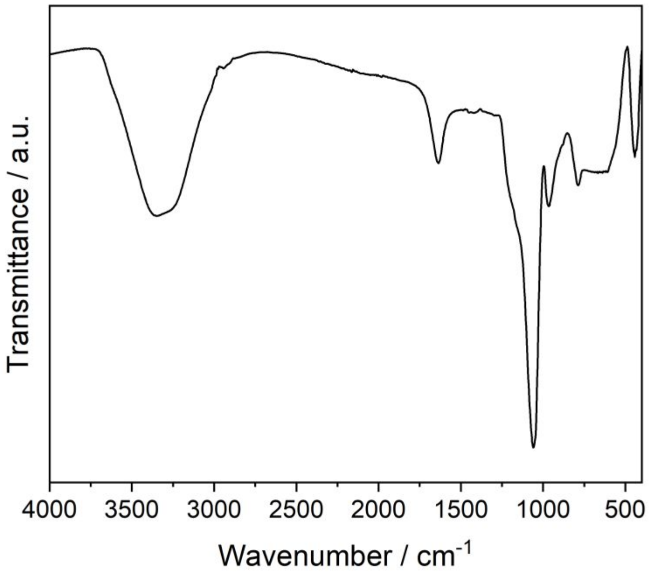

Figure S2. FTIR spectra of SiO_2 nanoparticles.

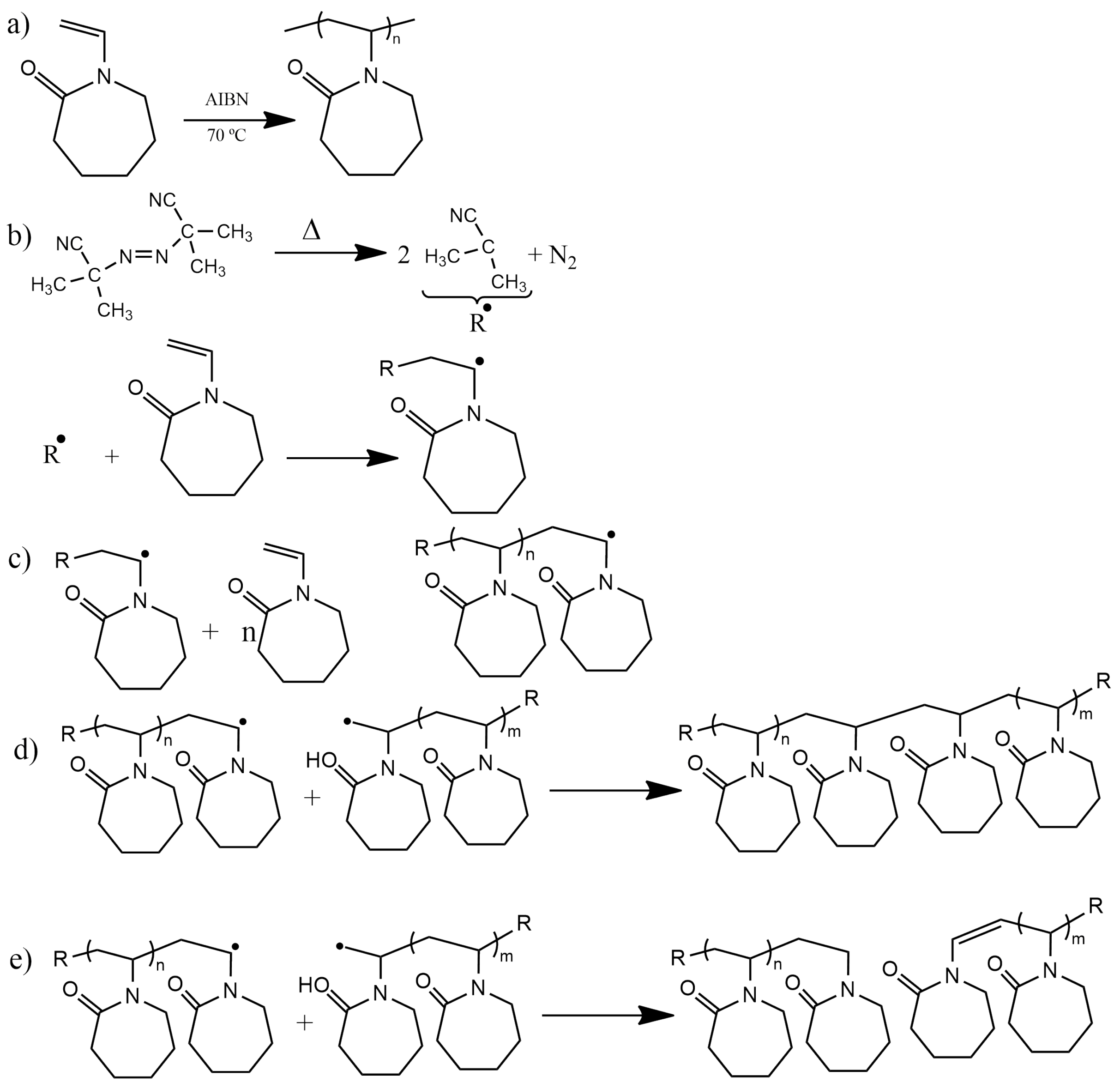

Figure S3. Reaction steps of PNVCL polymerization (a) with initiation step (b), propagation (c), termination by recombination (d), and by disproportionation (e)

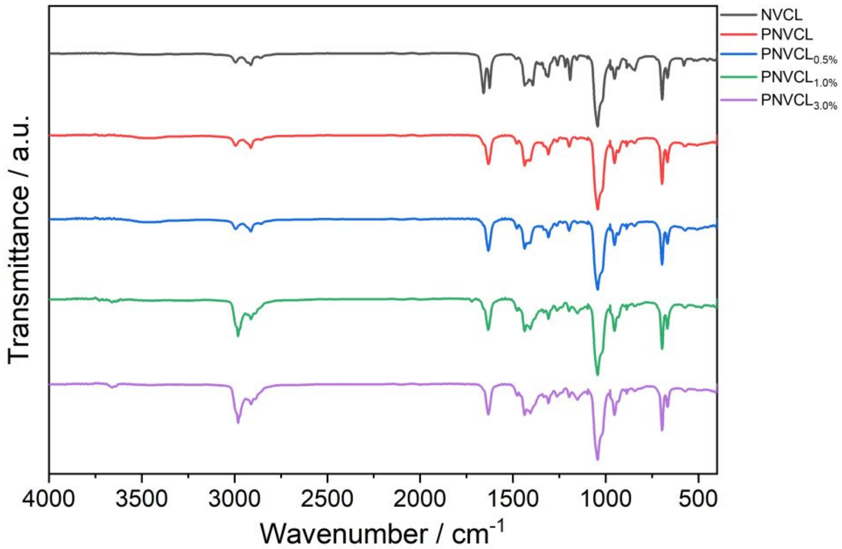

Figure S4. FTIR spectra of PNVCL products.

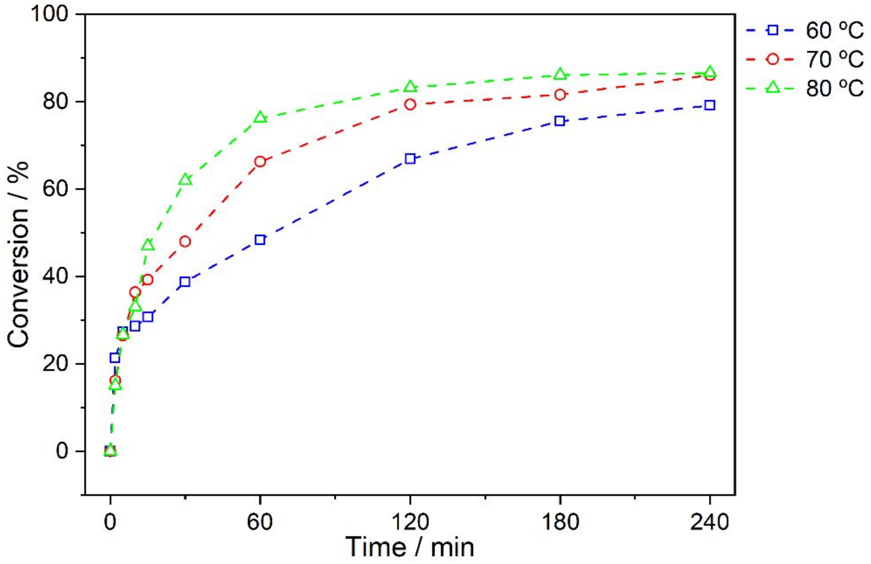

Figure S5. Dependence of conversion on temperature.

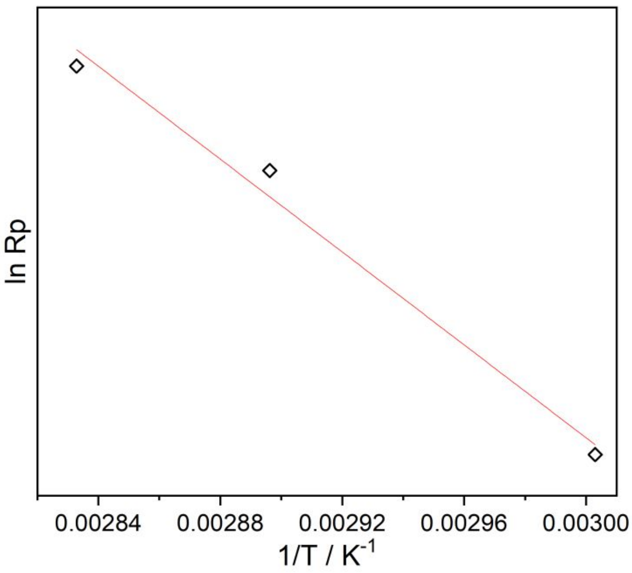

Figure S6. Arrhenius plot.

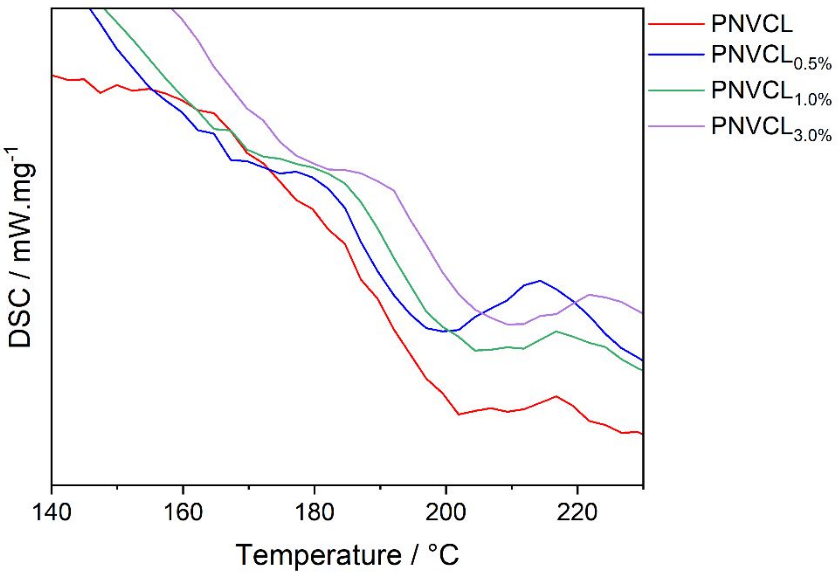

Figure S7. DSC curves.
